# Structural Variability Shows Power-Law Based Organization of Vowel Systems

**DOI:** 10.3389/fpsyg.2022.801908

**Published:** 2022-02-14

**Authors:** Menghan Zhang, Tao Gong

**Affiliations:** ^1^Institute of Modern Languages and Linguistics, Fudan University, Shanghai, China; ^2^Ministry of Education Key Laboratory of Contemporary Anthropology, Department of Anthropology and Human Genetics, School of Life Sciences, Fudan University, Shanghai, China; ^3^Key Innovation Group of Digital Humanities Resource and Research, Shanghai Normal University, Shanghai, China; ^4^School of Foreign Languages, Zhejiang University of Finance and Economics, Hangzhou, China; ^5^Google LLC, New York, NY, United States

**Keywords:** language universal, structural variability, self-organization, complex adaptive system, adaptive evolution

## Abstract

Speech sounds are an essential vehicle of information exchange and meaning expression in approximately 7,000 spoken languages in the world. What functional constraints and evolutionary mechanisms lie behind linguistic diversity of sound systems is under ongoing debate; in particular, it remains conflicting whether there exists any universal relationship between these constraints despite of diverse sounds systems cross-linguistically. Here, we conducted cross-linguistic typological and phylogenetic analyses to address the characteristics of constraints on linguistic diversity of vowel systems. First, the typological analysis revealed a power-law based dependence between the global structural dispersion and the local focalization of vowel systems and validated that such dependence was independent of geographic region, language family, and linguistic affiliation. Second, the phylogenetic analysis further illustrated that the observed dependence resulted from correlated evolutions of these two structural properties, which proceeded in an adaptive process. These results provide empirical evidence that self-organization mechanisms helped shape vowel systems and common functional constraints took effect on the evolution of vowel systems in the world’s languages.

## Introduction

Human beings can (re)organize hundreds of speech sounds to communicate with each other. Speech sounds in spoken languages undergo constant changes to achieve efficient and effective communications between speakers and listeners (Poulisse, 1997). Such communicative demands hence serve as the primary forces that drive the (re)organization of sound systems (de Boer, 2000). A recent comparison of approximately 7,000 spoken languages in the world has revealed a high degree of structural diversity in sound systems (Gordon, 2016). For example, the numbers of consonants in languages range from 6, as in Rotokas (ISO 639-3: roo), to 130, as in !Xóõ (ISO 639-3: nmn); and those of vowels range from 2, as in Zulgo (ISO 639-3: gnd), to 50, as in Aheu (ISO 639-3: thm) (Moran et al., 2014). Explaining the functional constraints and evolutionary mechanisms behind such diversity has become a fundamental goal of evolutionary linguistics (Dunn et al., 2011).

In practice, linguistic typologists aim to compare structural patterns in different linguistic components (e.g., sound systems) and summarize several universal tendencies therein. For example, as one of the pioneers in this field, Joseph Greenberg generalized 45 universal regularities [a.k.a., the Greenbergian universals (Croft et al., 2011)] against the syntactic and morphological diversities of the world’s languages (Greenberg, 1963). Following this work, other scholars have been focusing on discovering empirically co-occurring traits and ascribing observed universal tendencies to the general physical or socio-cognitive bases (or constraints) of humans (Shopen, 1985; Comrie, 1989; Futrell et al., 2015). Some of these scholars treat such universal tendencies as language-universal because the observed co-occurring traits are homogeneous across languages. By contrast, others suggest that some of these traits, such as the word order biases (Evans and Levinson, 2009; Dunn et al., 2011; Hammarström, 2016), are heterogeneous across languages and better interpreted as language-specific. Given that the majority of universal tendencies are basically statistical patterns or principles upon which different languages are constructed (Dunn et al., 2011), investigating the distributional trends of individual descriptive traits across languages and the dependencies among relevant traits can further clarify whether a universal tendency across languages is language-universal or language-specific.

Among the fundamental sound components of spoken languages (e.g., consonants, vowels, and tones), there have been growing and broad concerns on vowel systems because the typological structures and evolutions of vowel systems have been found to be subject to several well-studied functional constraints (de Boer, 2000; Zuidema and Boer, 2006; Chirkova and Gong, 2014). For example, the dispersion theory (Liljencrants and Lindblom, 1972) claims that the vowel systems are shaped predominantly by maximum vocalic distinctiveness in a perceptual domain, as a consequence of functional realization. This theory defines *dispersion* as a global structural property of a vowel system, which can be measured as the average inter-vowel perceptual distance of a system. Dispersion can help predict the occurrence frequencies of more (or less) favored structures of vowel systems worldwide (de Boer, 2000; Oudeyer, 2006; Zuidema and Boer, 2006). In addition, the Quantal theory (Stevens, 1972, 1989) states that the speech sounds easier or more reliable to produce are more common in the world’s languages, whereas those harder to produce are less so; in other words, the articulatory efforts serve as additional constraints on structural variability of sound systems (Bradlow, 1995; Lindblom and Maddieson, 2006). Integrating these two theories, the dispersion-focalization theory attempts to predict the structural formation of vowel systems based on competition between structural dispersion and focalization (Boë et al., 2002). *Focalization* here refers to a local structural property, which reflects the intra-vowel spectral salience of vowel sounds related to formant proximity in a perceptual domain. In addition to the theories on separate structural properties, other investigations of vowel systems from evolutionary linguistics focus on the interactions of structural properties (Kingston, 2007) and roles of iterated communications (de Boer, 2000; Oudeyer, 2006; Tamariz and Kirby, 2016).

However, it remains undetermined: whether there exists any dependence between global dispersion and local focalization; and if so, whether the dependence is language-universal or language-specific. To clarify these issues, this study conducted both synchronic and diachronic analyses on a worldwide phonetic database (Becker-Kristal, 2010). The database contains 532 individual samples of vowel systems covering 357 languages from 39 language families (see section “Materials and Methods”). As for global dispersion, we extended the dispersion theory by proposing a composite trait, effective dispersion estimate (Effective DE), to measure the structural crowdedness of a vowel system (see section “Materials and Methods”). As for local focalization, we adopted the local focalization estimate (FE) from the dispersion-focalization theory. We quantified the consistent dependence between Effective DE and FE using the whole dataset, and statistically examined the universality of this observed dependence. It is expected that if this dependence is language-universal, it should be ascertained to be independent of geographic region, language family, and even linguistic affiliation. Accordingly, we employed the statistical correlation and the stratification approach adopted in linguistic typology (Croft et al., 2011) to synchronically examine the dependence between Effective DE and FE in the language samples stratified by the major geographic regions and popular language families. Moreover, we applied the phylogenetic comparative methods to diachronically investigate the evolutionary correlation between these two structural properties given the linguistic affiliation. All these quantitative analyses help elaborate implicational universals of vowel systems and uncover any organizational behaviors behind those universals. The related findings also contribute to the discussion about the common functional constraints behind the evolution of sound systems in the world (Beckner et al., 2009; De Boer, 2015).

## Materials and Methods

### Formant Data Processing Based on Becker-Kristal’s Vowel System Database

This study compared the structures of vowel systems using the formant frequencies of vowels in different languages. All the data were extracted from the worldwide phonetic database of vowel systems established by Becker-Kristal (2010). According to his work, the formant frequencies of the vowels in the samples from the database were extracted from the recorded raw data using five elicitation methods: sustained isolated vowels, vowels embedded in isolated word lists, vowels embedded in words placed in carrier sentences, vowels embedded in words placed in meaningful sentences, and vowels pronounced as parts of speech flow such as conversations. The vowel systems of languages were also classified into six quantity-contrastive constituents: combined, mixed, long, short, uniform, and unknown. Due to different combinations of the elicitation methods and quantity-contrastive constituents, we treated the language samples with the same names (including language and dialect names) as distinct samples. We filtered out the samples that had incomplete first two formant frequencies of vowels, and thus obtained 532 samples covering 357 languages and dialects for the proposed analyses (see Becker-Kristal, 2010) for detailed descriptions of the corpus and other information of the recorded languages).

These 357 languages and dialects belonged to a total of 39 language families, in which nine families contained most of the samples, including: Indo-European (*N* = 233), Niger-Congo (*N* = 46), Afro-Asiatic (*N* = 31), Uralic (*N* = 31), Sino-Tibetan (*N* = 23), Nilo-Saharan (*N* = 19), Altaic (*N* = 17), Austronesian (*N* = 16), and Austroasiatic (*N* = 14). The geographic locations of the samples covered a total of eight world regions: Africa (Sample size *N* = 97), Europe (*N* = 223), Middle East (*N* = 14), Central South Asia (CS Asia; *N* = 36), East Asia (*N* = 84), Pacific (*N* = 19), South America (America S; *N* = 18), and North Central America (America NC; *N* = 41). The adoption of these eight regions followed a previous study (Creanza et al., 2015). Similar to many other language datasets, these samples are distributed in an unbalanced fashion across language families and geographic regions; for example, there are more languages in Europe, Africa, and East Asia than in South America and Pacific region. In addition, for some languages, due to cross-regional distributions of dialects, these languages appear simultaneously in different regions. Considering these, we conducted mixed-effects regression analyses to control for variances coming from languages, language families, and geographic regions to investigate the global patterns of vowel systems.

Furthermore, we extracted the first two formant frequencies of vowels from different sound systems which are frequently used to determine vowel quality. Linguistically, the first two distinguishable formants of vowels in the acoustic space refer to the high/low and front/back dimensions of the physical tongue position of the speaker (Becker-Kristal, 2010). Noting the articulatory activities in the physical space and the limited availability of the upper formant frequencies in the database, we did not use the effective second formant (a synthetic integration of the second, third, and higher formants) as in some studies (Liljencrants and Lindblom, 1972; de Boer, 2000).

### Psychoacoustic Conversion of Formant Data

Here, we transformed the formant frequencies of vowels into a psychoacoustic scale. This conversion is needed because the resolution of the human auditory system is determined by the critical band analysis following a non-linear Bark scale, such that high frequency sounds appear closer together than low frequency sounds (Liljencrants and Lindblom, 1972; de Boer, 2000). In practice, the Bark scale is widely adopted to stretch the vowel space where the auditory of human ears are most sensitive to frequency differences, and contracts the space where frequency differences are hard to perceive by human ears (Liljencrants and Lindblom, 1972; de Boer, 2000). For example, human ears have a high discrimination sensitivity in the low frequency domain (e.g., from 500 to 1,000 Hz), but a weak sensitivity in the high frequency domain (e.g., from 4,500 to 5,000 Hz).

The Bark scale that we used is defined as in Equation 1:


(1) $$B\,a\,r\,k=13\times \text{arctan}\,\left(0.00076\times F\,r\,e\,q\right)+3.5\times \text{arctan}\,{\left(\frac{F\,r\,e\,q}{7500}\right)}^{2}$$

### Effective Dispersion Estimate

To capture the global structure of vowel systems, we proposed the effective dispersion estimate (Effective DE), which integrates dispersion and articulatory space.

Dispersion is a systematic property of a vowel system (Liljencrants and Lindblom, 1972). It accumulates the reciprocal of total perceptual distances between each pair of vowels in a vowel system. It can be used to predict the most frequent vowel system(s) among languages and identify the most optimal one(s) in terms of clarity. Given a system of *n* vowels, the perceptual distance between pairwise vowels is measured as in Equation 2:


(2) $$d_{i,j}=\sqrt{{\left(F\,1_{i}-F\,1_{j}\right)}^{2}+{\left(F\,2_{i}-F\,2_{j}\right)}^{2}}$$

where F1*_*i*_*, F2*_*i*_*, F1*_*j*_*, F2*_*j*_* are the Bark values of the first and second formants of vowels *i* and *j*. And then, DE is calculated as the geometric average of the total perceptual distances for the whole vowel system. If vowels are very close to each other in a system, the distance among vowels is short and DE of the system is small.

Articulatory space is another systematic property of a vowel system measured by vowel space area. This space is also subject to the spatial distribution of vowels in a psychoacoustic domain. Accordingly, it can reflect the interaction between the vowel space area and structural dispersion of a vowel system. To quantify the articulatory space, we adopted the convex-hull approach (Liljencrants and Lindblom, 1972) in computational geometry to calculate the vowel space areas of vowel systems in different languages. In mathematics, a convex-hull of a set of X points in a Euclidean space is the smallest convex set that contains X. Convex-hull estimation is based on the relative positions of the vowels in a vowel space. It is more efficient and accurate in estimating the articulatory space area than the traditional method. In our study, we used the *convhull* function in Matlab R2015b to calculate the convex hull of each vowel system.

According to the original definition (Hammarström, 2016), a larger DE of a vowel system indicates that the vowels therein are more crowded in the vowel space, which seems counter-intuitive that a larger DE may also mean more spread out of vowels in the space. In our study, we proposed effective DE which was a modification of the original equation of DE (Hammarström, 2016). It integrated both the articulatory space and the average dispersion of a vowel system. It is obvious that given a specific number of vowels, the average dispersion of a vowel system is higher when the corresponding articulatory space is larger, and *vice versa* (see an example in Supplementary Figure 3). To eliminate the effects of acoustic space area, we defined *Effective DE* as the global dispersion (DE) divided by the acoustic space area, as in Equation 3. Like a normalization process, Effective DE measures the average dispersion estimate per unit area of a vowel system:


(3) $$E\,f\,f\,e\,c\,t\,i\,v\,e\,D\,E=\frac{D\,i\,s\,p\,e\,r\,s\,i\,o\,n\,e\,s\,t\,i\,m\,a\,t\,e\,\left(D\,E\right)}{A\,c\,o\,u\,s\,t\,i\,c\,s\,p\,a\,c\,e\,e\,s\,t\,i\,m\,a\,t\,e\,\left(A\,S\,E\right)}$$

### Focalization Estimate

Focalization is another structural property of a vowel system (Schwartz et al., 1997). FE can be measured as the relative perceptual distance between adjacent formants of vowels, which assigns a focal quality to each vowel. The concept of focal quality in vowel perception conceptually resembles the concept of “focal color” in color perception, which appears to be a cross-linguistic perceptual agreement (Heider, 1972; Gong et al., 2019). FE of a vowel system is the sum of the inverse-squares of the adjacent (F1 and F2) formant difference of each vowel. Accordingly, FE can indicate whether or not the vowel configuration produced by articulatory maneuvers is stable (Schwartz et al., 1997).

FE concerns two aspects: vowel quality and number of vowels. On the one hand, for example, FE of the vowel system /a, i, u/ is higher than that of /a, i, o/, because /u/ is more focal than /o/. Vowels with larger FE can be more easily and explicitly perceived by humans. On the other hand, FE of the vowel system /a, i, u, e/ is also higher than that of /a, i, u/, because the former system has one more vowel /e/ than the latter. Therefore, FE is a composite trait measuring the general focal quality of a vowel system. In our study, FE was measured as in Equation 4:


(4) $$F\,E=\sum_{i=1}^{n}\frac{1}{{\left(F\,2_{i}-F\,1_{i}\right)}^{2}}$$

### Linear Mixed-Effects Regression Models

To verify the power-law correlation, we logarithmically transformed (base 10) Effective DE and FE, and fitted a linear mixed-effects model (Quené and van den Bergh, 2008), which treated the log-transformed Effective DE as an independent variable and the log-transformed FE as a dependent one.

In the dataset, one language could have one or multiple (due to dialects) pairs FE and Effective DE values. Each language family contained multiple languages, so each family had multiple pairs of FE and Effective DE values. Similarly, a geographic region also contained multiple languages, and thus multiple pairs of FE and Effective DE values. These indicated a nested relation between language families, geographic regions, and language samples. Note that due to cross-regional distributions of dialects, some languages also had a not-fully crossed relation with some geographic regions, but such cases were very rare in our dataset. To investigate the general relationship between FE and Effective DE, we need to control for the variations coming from languages, language families, and geographic regions. Considering their nested relation, we incorporated two random intercepts in the mixed-effects model, namely language identity (ISO693) nested in language family (Family) and language identity nested in geographic region (Region).

The formula of the mixed-effects model is shown in Equation 5, in which the factor left of “∼” (meaning as a function of) is dependent and those right of “∼” are independent, and among the independent factors, those starting with “(1| “ are random intercepts, “/” means nesting (note that a random intercept “(1| A/B)” is equivalent to two random intercepts, “(1| A)” and “(1| A:B),” “:” means interaction):


(5) log(FE)∼log(EffectiveDE)+(1|Family/ISO693)+(1|Region/ISO693)

There are in total three groups of mixed-effects models. The first group contained one model that had the formula as in Equation 5. It directly tested whether there was a statistically significant power-law dependence between Effective DE and FE.

The second group of mixed-effects models were fitted to test whether the power-law dependence between Effective DE and FE was consistent in each of the eight geographic regions. Here, each mixed-effects model involved the random intercept of language identity nested in language family and was fitted on the subset data in each of the eight geographic regions.

The third group of mixed-effects models were fitted to test whether the power-law dependence between Effective DE and FE was consistent across language families. Here, our analysis focused only on the nine major language families each having more than 10 samples in the database. Many of the families have relatively large numbers of languages and dialects and large speaker population sizes (Hammarström, 2016). Each model in this group involved the random intercept of language identity nested in geographic region and was fitted on the subset data in each of the nine language families.

Although the first mixed-effects model covered the whole dataset, together with the between geographic region and between language family analyses, the second and third groups of mixed-effects models provided additional evidence on whether the identified power-law dependence between Effective DE and FE from the whole dataset was independent of specific geographic regions and language families. The results of the second and third groups of mixed-effects models are shown in Supplementary Tables 1, 2 and Supplementary Figures 1, 2 in Supporting Information.

Compared to simple linear models, mixed-effects models allow for simultaneous consideration of multiple covariates, while keeping the between-individual (family and region) variance under statistical control (Baayen, 2008). Although maximal random effect structures involving random slopes are theoretically desirable (Barr et al., 2013) and have been applied in recent individual difference studies (e.g., Protopapas et al., 2007), such complicated models were not pursued here in consideration of practical constraints on model convergence (Bates et al., 2015). In our study, the mixed-effects models were implemented using the *lme4* (Bates et al., 2015) and *lmerTest* (Kuznetsova et al., 2017) packages in R.

### Phylogeny of 33 Indo-European Languages

The posterior tree distributions for Indo-European lexical dataset were inferred using a Bayesian Markov Chain Monte Carlo approach implemented in the program *BayesPhylogenies 1.1* (Pagel and Meade, 2004).^1^ Based on the lexical dataset in the previous work (Bouckaert et al., 2012), we used a two-state covarion model of lexical evolution, allowing cognate sets to change with heterogeneous rates along the tree. We ran the data for 12 million generations as plots of the critical parameters and constrained the root of the tree as three old languages of Hittite, Tocharian A and B. This was sufficient for convergence. We sampled the trees from the posterior every 10 thousand generations and set the parameter of burn-in to two million generations. Accordingly, these initial trees were discarded leaving a total of 1,000 trees in the Bayesian posterior sample and the consensus tree was constructed using the program *BayesTrees V1.4*.^1^

Then, we pruned the posterior tree samples and the consensus tree according to the intersection between the language samples in our database and those in the previous work (Bouckaert et al., 2012). We thus obtained 166 language samples covering the 33 IE languages which had the same ISO693-3 codes and close geographic regions. For the language samples with the same ISO693-3 codes, we took the number of individual speakers in each IE language sample as the weight and calculated the weighted average values of Effective DE and FE. Consequently, we got 33 pairs of Effective DE and FE and pairs of their logarithmically transformed (base 10) values for the 33 IE languages, respectively (see Supplementary Dataset 2).

### Phylogenetically Independent Contrast

Phylogenetic effect (*a.k.a* Galton’s problem; Naroll, 1961, 1965) may cause false positives to the relationship between the two features, because related languages tend to share similar characteristics inherited from their common ancestral language, and accordingly, any dependence or relationship could be driven by language inheritance. In contrast to the stratification analysis, PIC provides an alternative solution to Galton’s problem, which can infer the actual independent changes after controlling for the phylogenetic relationship among languages (Levinson and Gray, 2012).

We adopted the approach of phylogenetically independent contrast (PIC) to examine the dependence between EDE and FE, and that between log_10_(Effective DE) and log_10_(FE), with control for effects of the Indo-European (IE) language phylogeny. Accounting for the phylogenetic uncertainties, the 1,000 Bayesian posterior tree samples of 33 IE languages were used in PIC. The PIC is implemented in *Continuous* package of *BayesTraits V3*.^1^ We used 100 stepping-stones and 1,000 iterations per stone to estimate the values of marginal likelihood. Total iteration was set as 1,010,000 and the first 10,000 was Burn-in. To evaluate the significance of the evolutionary correlation, we used Bayes Factor to compare the independent and dependent model for Effective DE and FE, and between their log-transformed (base 10) values, respectively. To be specific, the correlation coefficient in the independent model was set to zero, indicating that the two structural properties were not correlated.

### Phylogenetic Generalized Least Squares Analysis

Alternative to the PIC method, we employed Phylogenetic Generalized Least Squares (PGLS) analysis to investigate the mechanism underlying this correlated evolution through multivariate Brownian motion (BM) and Ornstein-Uhlenbeck (OU) processes. The multivariate BM process can encompass the situation where each trait evolves independently of one another, yet it can also describe the situation where traits evolve in a correlated way. The multivariate OU process can model stabilizing selection pressures on correlated evolution of two continuous traits toward a fitness optimum on an adaptive landscape. Here, we used the consensus tree of 33 IE languages for the PGLS analysis, in which we examined the correlated evolution of Effective DE and FE fitting multivariate BM or OU models. In particular, we first created the phylogenetic correlation structures using the Brownian motion (corBrownian) and Ornstein-Uhlenbeck (corMartins) models of character covariance, which were implemented using *ape* package version 5.4-1 and its dependencies (Felsenstein, 1985; Paradis et al., 2004; Revell, 2010; Paradis, 2012). Then, we adopted the *gls* function in *nlme* package (Pinheiro et al., 2021) to run the PGLS analysis, in which Effective DE was treated as a function of FE (Paradis, 2012). After that, we adopted the likelihood ratio test to statistically compare the BM model and the OU model, using the *anova* function in R.

## Results

### The Dependence Between Effective Dispersion Estimate and Focalization Estimate of Vowel Systems

**FIGURE 1:**
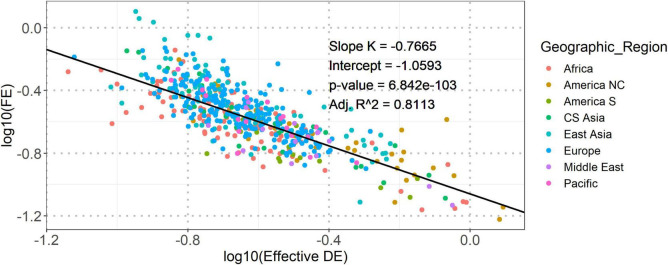
The dependence between logarithmically transformed Effective DE and FE. The solid line shows the best fit of linear relationship between the two logarithmically transformed (base 10) structural properties. The slope (*K*), intercept, and corresponding *p*-value of the linear fitting model are shown in the inline legends. “Adj. *R*^2^” is the adjusted *R*^2^. Points in both figures are colored according to geographic regions.

At first, we applied the Spearman’s correlation analysis to investigate the relation between Effective DE and FE based on all 532 vowel systems from the database. The result showed a significant negative correlation between the global and local structural properties (Spearman’s *rho* = −0.8427, *p*-value = 0.0). To further verify this correlation, we fitted a linear mixed-effect model having two random intercepts (language identity nested in language family and language identity nested in geographic region) on the whole dataset. The result reported a good linear fitting [adjusted *R*^2^ = 0.8113, the adjusted *R*^2^ measures the proportion of variance explained by both fixed and random (if any) factors of the model, implemented by the r_squaredGLMM function from *MuMIn* package (Bartoń, 2020)] between the transformed properties (see Figure 1), suggesting a power-law dependence between Effective DE and FE. Power-law is a signature of specific stochastic process in a complex system (Kello Kello et al., 2010). Thus, our observation suggested that the vowel systems in the world’s languages should be shaped by dynamic structural (re)arrangement at both global and local levels.

### The Dependence Across and Within Geographic Regions

Our language samples were distributed in eight major geographic regions: Africa, Middle East, Europe, East Asia, Central-South Asia (CS Asia), and South America (America S), North-Central America (America NC), and Pacific. Many of these regions contain many languages and/or dialects (Hammarström, 2016).

**FIGURE 2:**
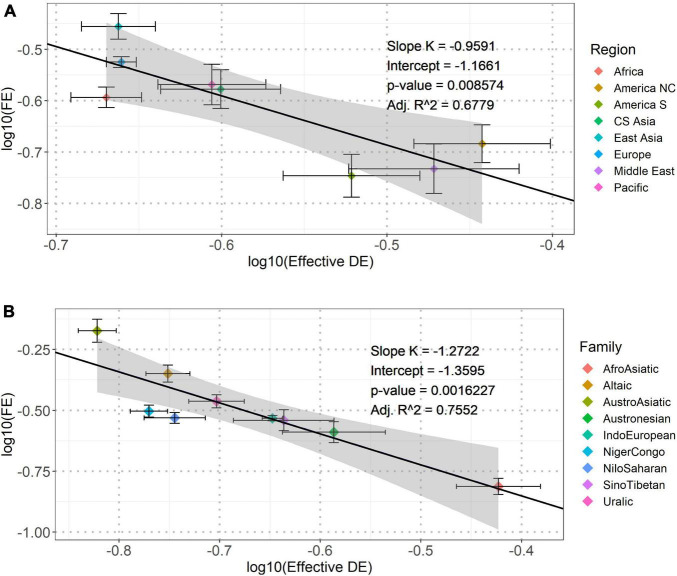
The linear regression plots of [logarithmic transformation (base 10)] Effective DE against FE across the eight geographic regions **(A)** and the nine major language families **(B)**. The solid line in each panel represents the best fitting linear regression model between log_10_(Effective DE) and log_10_(FE). The slopes (*K*), intercept, corresponding *p*-values and adjusted *R*^2^ (Adj. *R*^2^) are shown in the inline legends. The shaded area mark standard errors. For each data point, the two error bars indicate standard errors of log_10_(Effective DE) and log_10_(FE) in each geographic region or major language family.

We investigated the consistency of the power-law dependence stratified by the geographic regions. As for inter-regional comparison, we first obtained the mean values of Effective DE and FE in each of the eight geographic regions, and then, fitted a linear regression between each pair of the logarithmically transformed (base 10) values. This model showed that the average FE also had a significant negative dependence on those of Effective DE across all geographic regions (Figure 2A). As for intra-regional comparison, significant negative correlations between Effective DE and FE existed in each of the eight geographic regions (see Supplementary Table 1 and Supplementary Figure 1 in the Supporting Information for the results of the mixed-effects models). These inter- and intra-regional comparisons collectively evidenced that the power-law dependence between EDE and FE was not geographically specific.

### The Dependence Across and Within Language Families

In addition to geographic regions, we also examined the consistency of the power-law dependence across and within language families. Our analysis focused on nine major language families each having more than 10 samples in the database. Resembling the analyses across geographic regions, we first obtained the mean Effective DE and FE in each of the nine language families, and then, fitted a linear regression between each pair of the logarithmically transformed (base 10) values. The results are shown, respectively, in Figure 2B above (see Supplementary Table 2 and Supplementary Figure 2 in the Supporting Information for the results of the mixed-effects models). These inter- and intra-regional comparisons collectively evidenced that the power-law dependence between Effective DE and FE was lineage-general across languages.

### Correlated Evolution of Effective Dispersion Estimate and Focalization Estimate in Indo-European Languages

To test the correlated evolution of Effective DE and FE, we took the Indo-European (IE) languages as an example and applied the phylogenetic comparative methods to the phylogeny of 33 IE languages derived from an early study (Bouckaert et al., 2012) (see section “Materials and Methods”). As shown in the previous sections, the language samples in the dataset we investigated were unbalanced and there were repeated measures of the same language. Therefore, although the samples in the dataset did cover many language families, when mapping samples to languages of families to construct phylogenetic trees, the Indo-European family became the only family containing sufficiently many (> 30) samples. Accordingly, we built phylogenetic trees based on samples only from this family.

Considering phylogenetic uncertainties, we performed the PIC method based on 1,000 Bayesian posterior tree samples of 33 IE languages. The phylogenetic results revealed a strong-negatively correlated evolution between the raw and log-translated values of Effective DE and FE of the vowel systems (see Table 1).

**TABLE 1:** The log-likelihoods for the dependent and independent models of correlated evolution between the raw and log-transformed Effective DE and FE reported by the PIC method.

Name	Model	Log likelihood	rho ± sd	Log_10_ Bayes factor
FE ∼ Effective DE	Independent	45.0594	0.00	–
	Dependent	55.5306	−0.6760 ± 0.0086	20.9424
log10(FE) ∼ log10(Effective DE)	Independent	26.9014	0.00	–
	Dependent	41.2260	−0.7592 ± 0.0082	28.6492

*Log10 Bayes factor indicates the relative support for the dependent model over the independent one. A value below 2 suggests a weak support, one over 2 a positive support, one between 5 and 10 a strong support, and one over 10 a decisive support. Log likelihood for each model is a marginal.*

The statistical results of the Phylogenetic Generalized Least Squares (PGLS) analysis showed a correlated evolution of Effective DE and FE following the OU process (Butler and King, 2004; see Table 2). It further suggested that the correlated evolution follow an adaptation process under the stabilizing selection pressures. This is compatible with the results of our synchronic comparisons: The evolution of vowel systems is a dynamic process of adaptive organization of their structures and optimization capacities.

**TABLE 2:** The log-likelihoods for the Brownian motion and Ornstein-Uhlenbeck models for the raw and log-transformed Effective DE and FE reported by the PGLS method.

Name	Model	Log likelihood	β ± sd	Likelihood ratio (*p*-value)
FE ∼ Effective DE	Brownian motion	37.7372	−0.9331 ± 0.1598	–
	Ornstein-Uhlenbeck	43.5307	−0.8322 ± 0.1669	21.5870 (*p* < 0.0001)
log10(FE) ∼ log10(Effective DE)	Brownian Motion	36.5835	−0.9177 ± 0.0994	–
	Ornstein-Uhlenbeck	44.9317	−0.8643 ± 0.1157	11.6283 (*p* = 6e-04)

## Discussion

Clarifying whether an observed co-occurring trait across languages is language-universal or language-specific is important to understand the functional constraints behind apparent linguistic diversity. By means of statistical approaches and phylogenetic comparative methods, this study discovered a power-law (with a negative exponent) dependence between the global and local structural properties (indicated, respectively, by effective dispersion, Effective DE, and local focalization, FE) of vowel systems and demonstrated that such dependence was a language universal tendency, being independent of geographic region, language family, and linguistic affiliation. We further revealed that the correlated evolutions of these structural properties tended to proceed in an adaptive process.

Power-law is a widely adopted, crucial indicator of self-organization property of complex adaptive systems (Bak et al., 1987; Bak, 2013). The observed power-law dependence between Effective DE and FE thus becomes a hallmark of the self-organization property of vowel systems in human languages. In such system, self-organization can be viewed as a dynamic equilibria of structural optimization (de Boer, 2000; Oudeyer, 2006). “Optimization” here refers to constant structural (re)adjustment of human speech under the constraints spanning from production and perception of vowels. It is in line with the OU process identified from Effective DE and FE and from their logarithmically transformed (base 10) values. In this sense, the observed power-law dependence indicates that vowel systems could dynamically optimize their structural layouts in different ways (e.g., adding focal vowels, merging adjacent vowels in the acoustic/perceptual space, or rearranging the shape of vowel space). For example, given a relatively stationary vowel space area, a vowel system with more vowels tends to decrease the acoustic distinctiveness among its vowels and simultaneously increase the structural crowdedness. Decrease in acoustic distinctiveness would not benefit fundamental communicative demands of human speech, because closer vowels raise more auditory confusions. Then, to maintain efficient communications, the vowel system begins to (re)arrange its internal structure to obtain vowels with focal qualities.

Language is constantly changing. In terms of vowel systems, languages tend to accumulate inconspicuous changes in typological structures over time, such as numerous acoustic variations of individual vowels in a speaker population (Wang, 1969; Fox, 1982), in order to achieve efficient speech understanding and high speech intelligibility under a variety of conditions and disturbances. Such slow accumulation of structural changes continues until a threshold is reached, and then, the whole system might be rapidly restructured (Goodenough, 1992). Here, the threshold is similar to the critical point in self-organizing system in physics, which was first discovered in self-organized criticality (Per Bak and Wiesenfeld, 1987).

As shown in Figures 1, 2, the range of the slopes of the linear fitting models was [-0.77, -1.27], indicating that the structural (re)organization of vowel systems could probably follow a power law with a slope in a linear axis being around -1.0. Such power-law is also called Zipf’s law (Zipf, 1949). It was first observed by linguist George Zipf in the relationship between word use frequency and word rank. He theorized that such a power-law distribution was due to the tendency to communicate efficiently with least effort, which reflects the general principle of least effort in information science and evolutionary biology. This principle states that an information seeking client (humans, animals, or well-designed machines) tends to use the most convenient search method, in the least mode available, and such information seeking behavior tends to stop once minimally acceptable results are obtained. A typical characteristic of a system showing the principle of least effort is a power-law (in many cases, a Zipf’s law) distribution of system components (Kello Kello et al., 2010). Similar to the case of least effort in word use, human languages also tend to change the structures of their components [e.g., adjusting vowel systems or minimizing the dependence length in syntax (Futrell et al., 2015)] to fulfill communicative demands from language users. Therefore, the observed Zipf’s law like dependence and the identified adaptive evolution of the dependence in our study imply that the structural (re)organization of vowel systems could follow the principle of least effort.

In addition, the large slope variance of the linear fitting models reflected different structural variability of vowel systems across the geographic regions (mean = −0.7739, *SD* = 0.1255) and the major language families (mean = −0.8350, *SD* = 0.3941). These variances could be due to two factors. The first one is the intrinsic linguistic typology of vowel systems, which concerns not only the sizes of vowel systems but also the qualities of their vowels. The second one is the specific evolutionary histories of different languages. Humans are the primary carriers of languages. Evolutionary processes of different languages can be attributed to specific demographic activities in local regions and cultural developments within or between language families. Then, various attributions trigger diverse performances of structural organization of languages (Hammarström, 2016). Despite of such diversity, the power-law dependence remains universal across geographic regions and language families.

Traditional linguistic typological investigations are based primarily on correlation analysis of synchronic patterns (Hammarström, 2016). Although synchronic patterns are reflex of diachronic changes (Ladd et al., 2015), synchronic patterns alone are insufficient to reconstruct the evolutionary histories of languages, and our investigation of the diachronic changes of Effective DE and FE is also subject to such limitation. Fortunately, the phylogenetic comparative approaches helped identify the correlated evolutions of the structural properties of vowel systems (see Supplementary Materials). As expected, the synchronic and diachronic results in our study were consistent with each other. They not only complemented the universality of the power-law dependence, but also provided valuable insights on many important questions about linguistic diversity and language evolution (Greenhill, 2015; Hammarström, 2016).

## Data Availability Statement

The original contributions presented in the study are included in the article/Supplementary Material, further inquiries can be directed to the corresponding author/s.

## Author Contributions

MZ and TG designed the study, interpreted and discussed the results, and wrote the manuscript. MZ implemented the model. Both authors contributed to the article and approved the submitted version.

## Conflict of Interest

TG was employed by the company Google LLC. The remaining author declares that the research was conducted in the absence of any commercial or financial relationships that could be construed as a potential conflict of interest.

## Publisher’s Note

All claims expressed in this article are solely those of the authors and do not necessarily represent those of their affiliated organizations, or those of the publisher, the editors and the reviewers. Any product that may be evaluated in this article, or claim that may be made by its manufacturer, is not guaranteed or endorsed by the publisher.

## Footnotes

1
https://github.com/AndrewPMeade/
