# Supplementary material for: Structural Variability Shows Power-Law Based Organization of Vowel Systems
Source: Front Psychol. 2022 Feb 14;13:801908. doi: 10.3389/fpsyg.2022.801908 (PMC8882920; doi:10.3389/fpsyg.2022.801908)
Supplement: Supplementary file 3 [file Data_Sheet_3.pdf]

Supporting Information

Structural variability shows power-law based organization of vowel systems

Menghan Zhang, Tao Gong

Supplementary files (see https://github.com/gtojty/PowerLawVowelSystems)

Dataset 1. 532 language samples used in our study.

Dataset 2. 33 IE languages used in our study.

Matlab codes for correlation analyses and model fitting.

R codes for phylogenetic analyses.

R codes for power law fitting curves based on the whole dataset and subsets divided by language families and geographic regions.

Supporting Tables

Table S1. The mixed-effects analysis results between log-transformed (base 10) Effective DE and FE of the language samples in each of the eight geographic regions (a linear regression model is used if a mixed-effects model does not converge). *p*-value < 0.05 indicates significance. Fitted curves are shown in Fig S1. "Adj. R^2" is the conditional R^2 measuring the proportion of variance explained by both fixed and random factors.

**Region**	**Sample size**	**Slope**	**Intercept**	***p*-value**	**Adj. R^2**
Africa	97	-0.7192	-1.0856	2.44e-14	0.7288
America NC	41	-0.7816	-1.0567	2.42e-13	0.9189
America S	18	-0.7661	-1.147	0.0005	0.8836
CS Asia	36	-0.871	-1.1085	2.71e-13	0.8986
East Asia	84	-0.6385	-0.8786	4.16e-13	0.8084
Europe	223	-0.8468	-1.0784	1.57e-41	0.7209
Middle East	14	-0.5919	-0.9953	0.0020	0.8017
Pacific	19	-0.9764	-1.1602	2.89e-05	0.6393

Table S2. The mixed-effects analysis results between log-transformed (base 10) Effective DE and FE of 532 language samples in the nine language families (a linear regression model is used if a mixed-effects model does not converge). *p*-value < 0.05 indicates significance. The shaded row does not reach to a significant level. Fitted curves are shown in Fig S2. "Adj. R^2" is the conditional R^2 measuring the proportion of variance explained by both fixed and random factors.

**Language family**	**Sample size**	**Slope**	**Intercept**	***p*-value**	**Adj. R^2**
Indo-European	233	-0.8424	-1.0782	2.02e-53	0.7596
Niger-Congo	46	-0.8308	-1.0897	1.54e-06	0.6727
Afro-Asiatic	31	-0.6417	-1.0859	2.39e-09	0.7824
Uralic	31	-0.9461	-1.0747	8.71e-05	0.8057

Sino-Tibetan	23	-0.6748	-1.0129	6.00e-07	0.9015
Nilo-Saharan	19	-0.3912	-0.8224	0.0180	0.276
Altaic	17	-0.7536	-0.9215	0.0795	0.3439
Austronesian	16	-0.6373	-0.9635	0.0008	0.5494
Austro-Asiatic	14	-1.7965	-1.6492	0.0037	0.4978

Supporting Figures

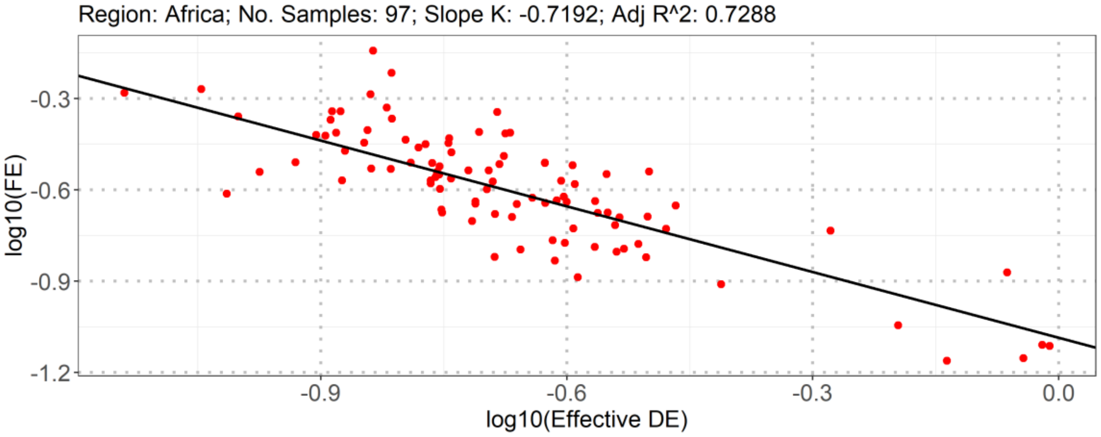

(a)

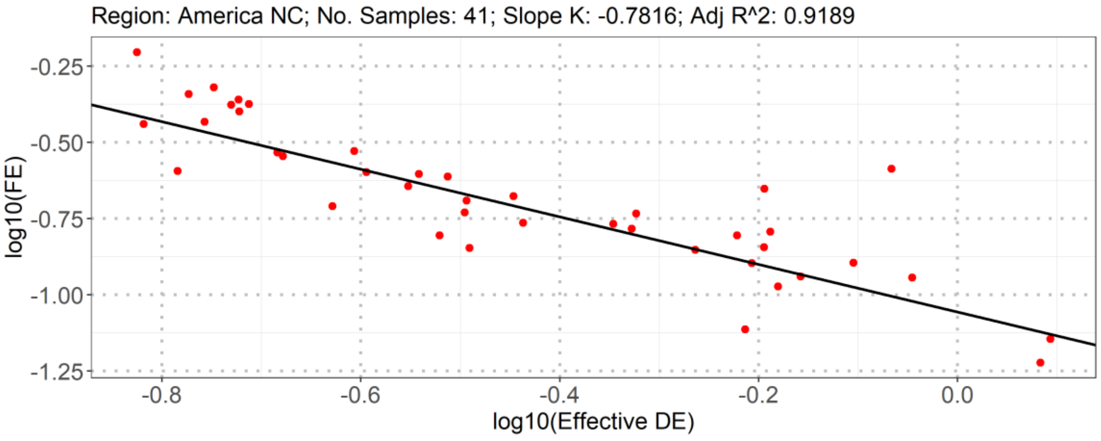

(b)

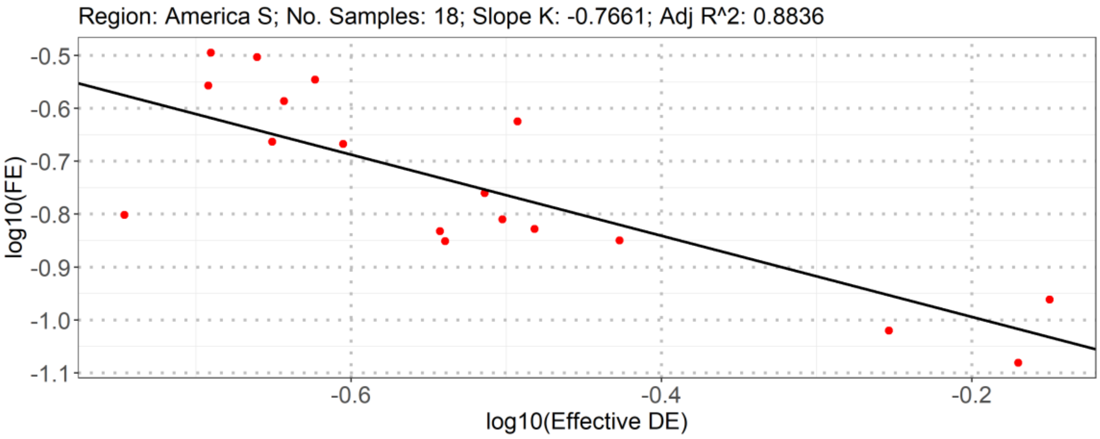
Region: America S; No. Samples: 18; Slope K: -0.7661; Adj R^2: 0.8836
log10(FE)
-0.5
-0.6
-0.7
-0.8
-0.9
-1.0
-1.1
-0.6
-0.4
-0.2
log10(Effective DE)

(c)

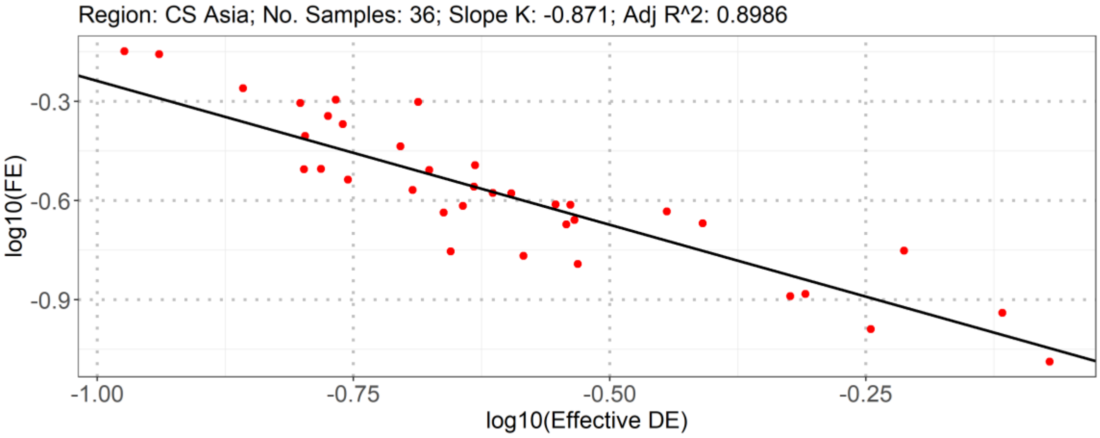
Region: CS Asia; No. Samples: 36; Slope K: -0.871; Adj R^2: 0.8986
log10(FE)
-0.3
-0.6
-0.9
-1.00
-0.75
-0.50
-0.25
log10(Effective DE)

(d)

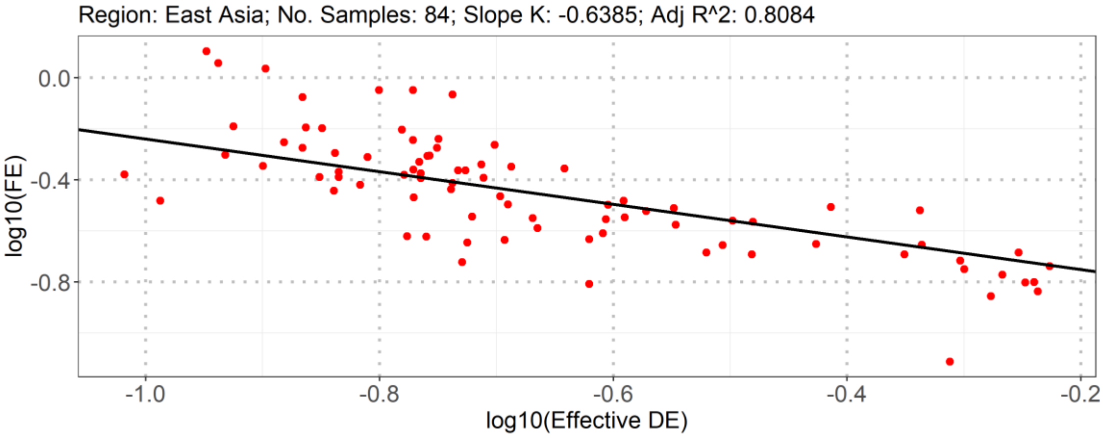
Region: East Asia; No. Samples: 84; Slope K: -0.6385; Adj R^2: 0.8084
log10(FE)
0.0
-0.4
-0.8
-1.0
-0.8
-0.6
-0.4
-0.2
log10(Effective DE)

(e)

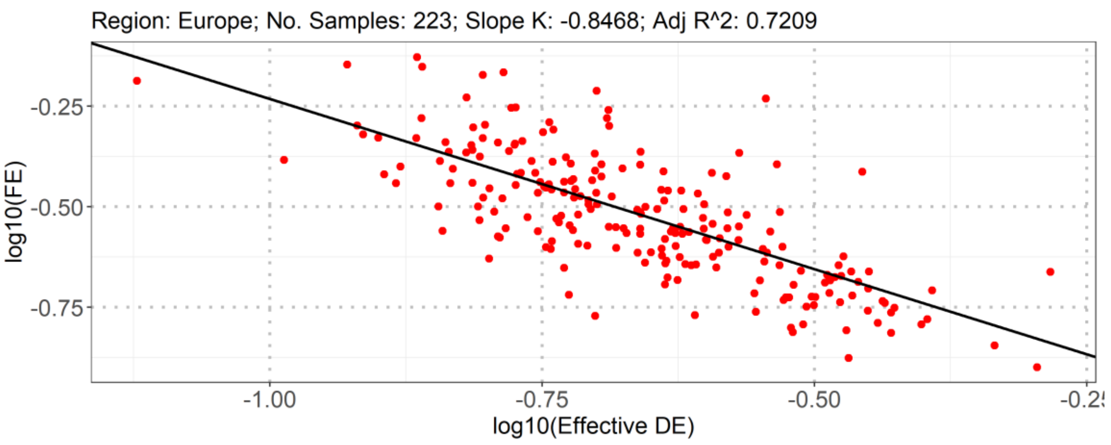

(f)

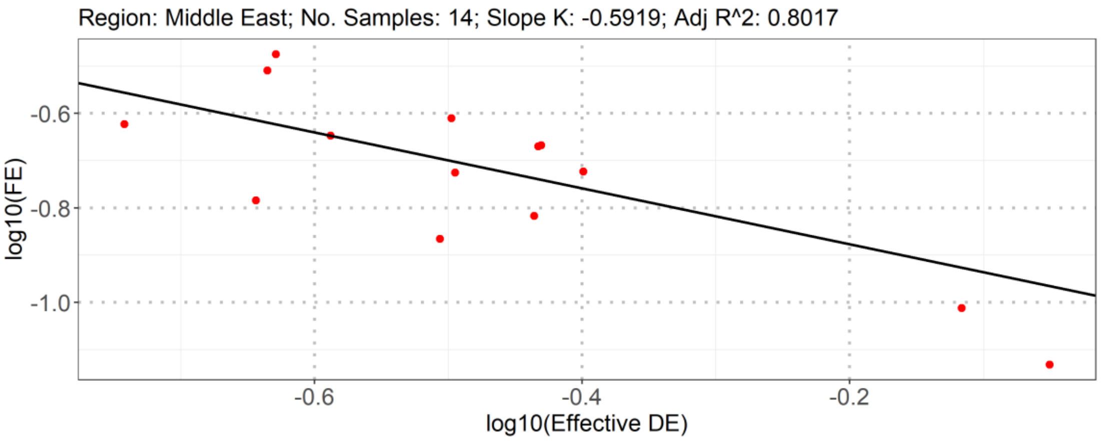

(g)

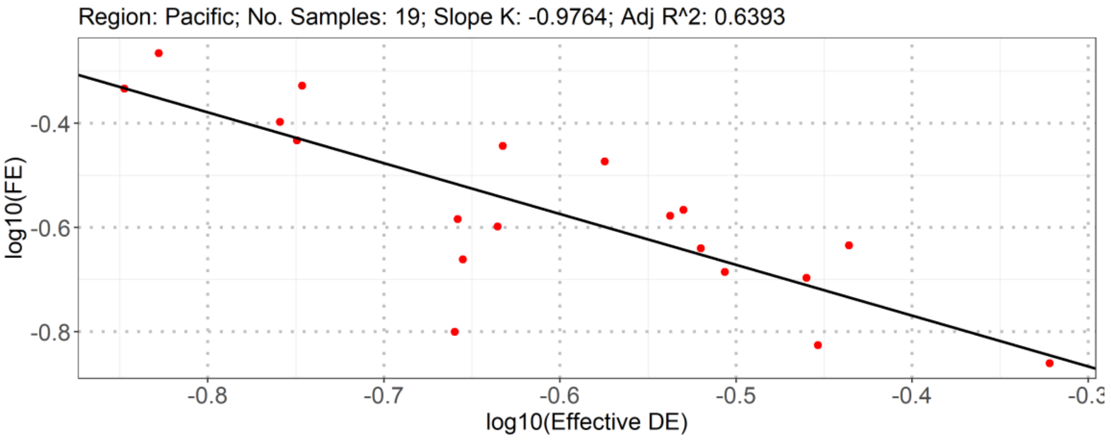

(h)

Fig S1. The mixed-effects linear regression plots of log-transformed (base 10) EDE against FE in the eight geographic regions. The solid line in each panel denotes the best fitting linear regression curve. “Adj. R^2” is the conditional R^2 measuring the proportion of variance explained by both fixed and random factors.

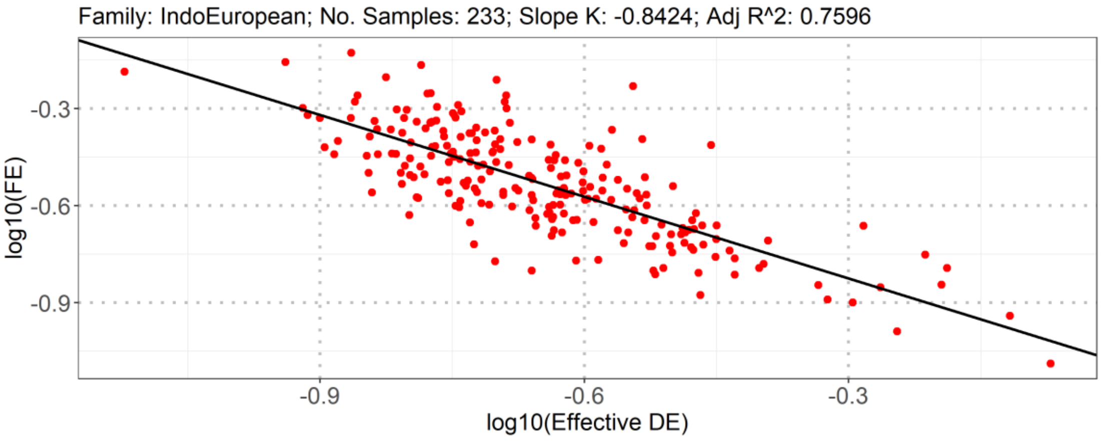

Family: IndoEuropean; No. Samples: 233; Slope K: -0.8424; Adj R^2: 0.7596
-0.3
-0.6
-0.9
log10(FE)
-0.9
-0.6
-0.3
log10(Effective DE)

(a)

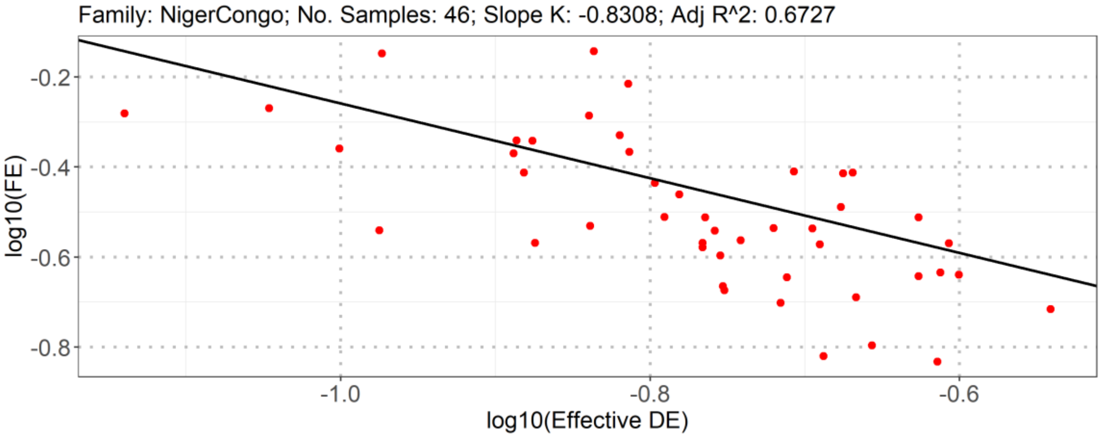

Family: NigerCongo; No. Samples: 46; Slope K: -0.8308; Adj R^2: 0.6727
-0.2
-0.4
-0.6
-0.8
log10(FE)
-1.0
-0.8
-0.6
log10(Effective DE)

(b)

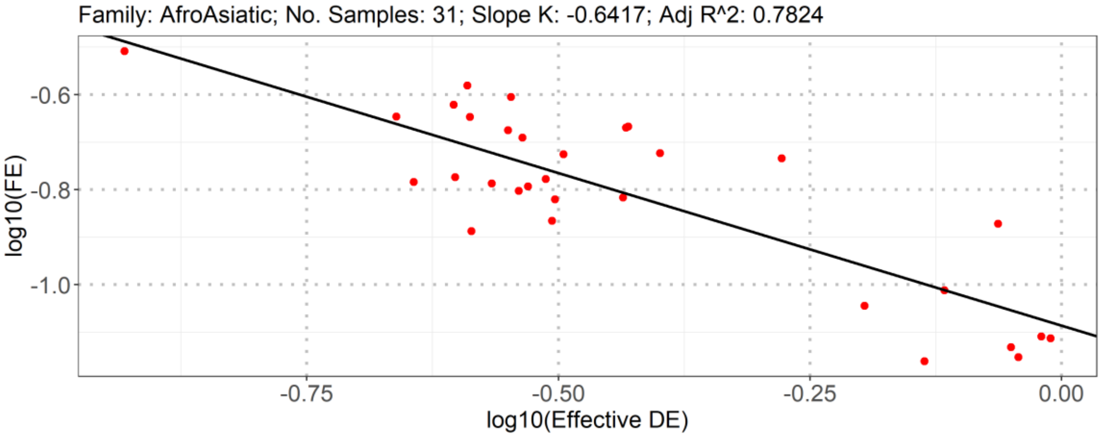

Family: AfroAsiatic; No. Samples: 31; Slope K: -0.6417; Adj R^2: 0.7824
-0.6
-0.8
-1.0
log10(FE)
-0.75
-0.50
-0.25
0.00
log10(Effective DE)

(c)

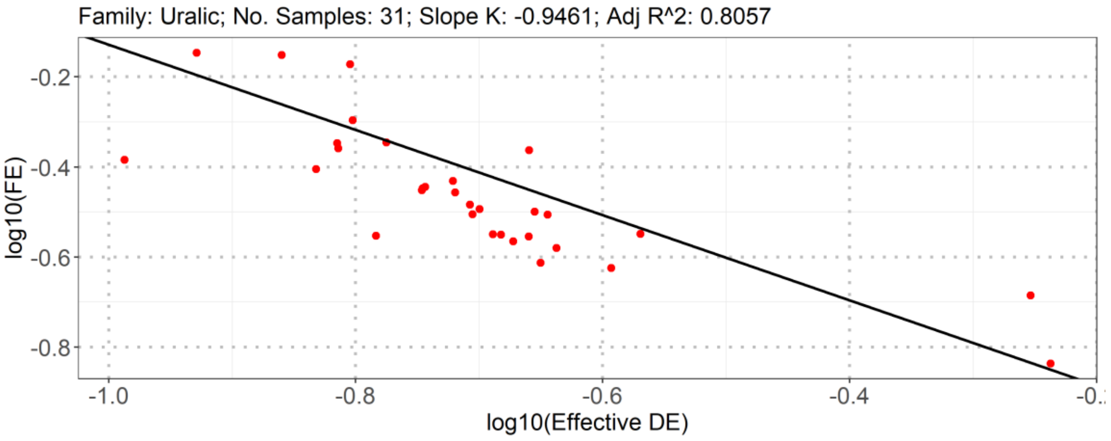
Family: Uralic; No. Samples: 31; Slope K: -0.9461; Adj R^2: 0.8057
-0.2
-0.4
-0.6
-0.8
log10(FE)
-1.0
-0.8
-0.6
-0.4
log10(Effective DE)

(d)

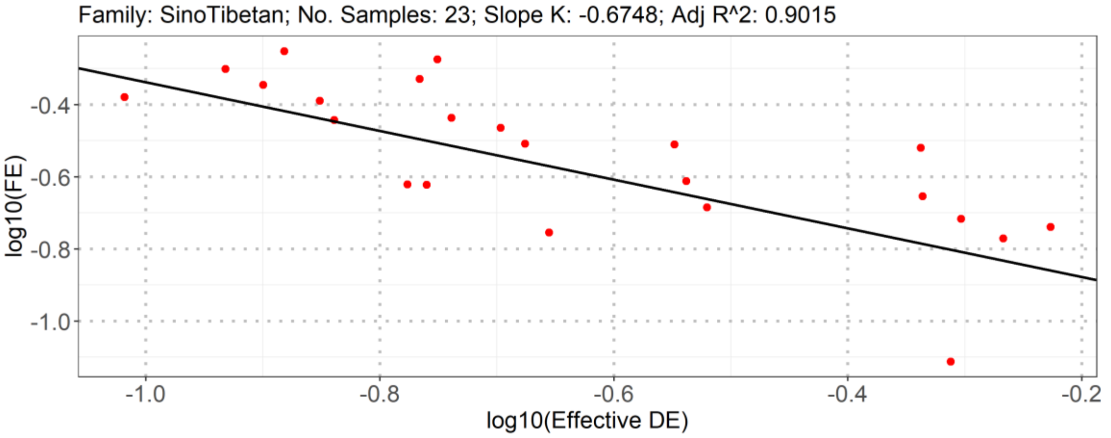
Family: SinoTibetan; No. Samples: 23; Slope K: -0.6748; Adj R^2: 0.9015
-0.4
-0.6
-0.8
-1.0
log10(FE)
-1.0
-0.8
-0.6
-0.4
-0.2
log10(Effective DE)

(e)

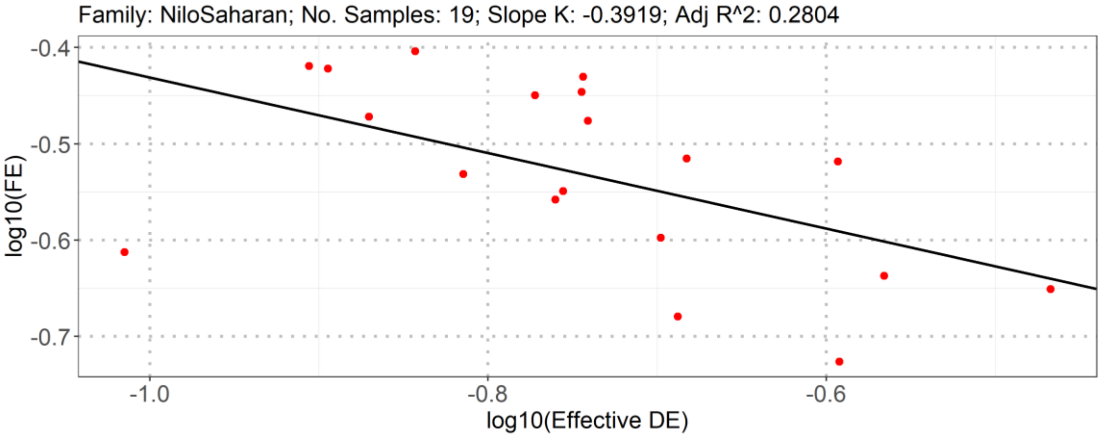
Family: NiloSaharan; No. Samples: 19; Slope K: -0.3919; Adj R^2: 0.2804
-0.4
-0.5
-0.6
-0.7
log10(FE)
-1.0
-0.8
-0.6
log10(Effective DE)

(f)

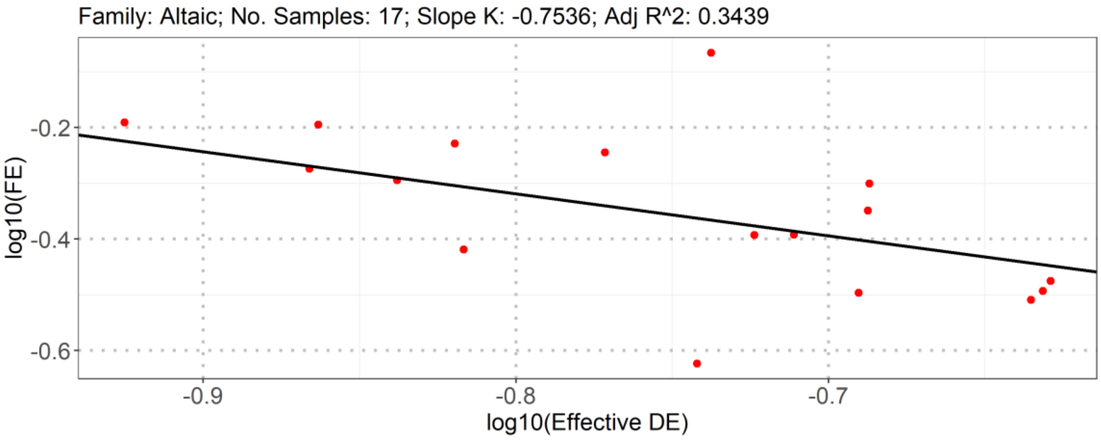

(g)

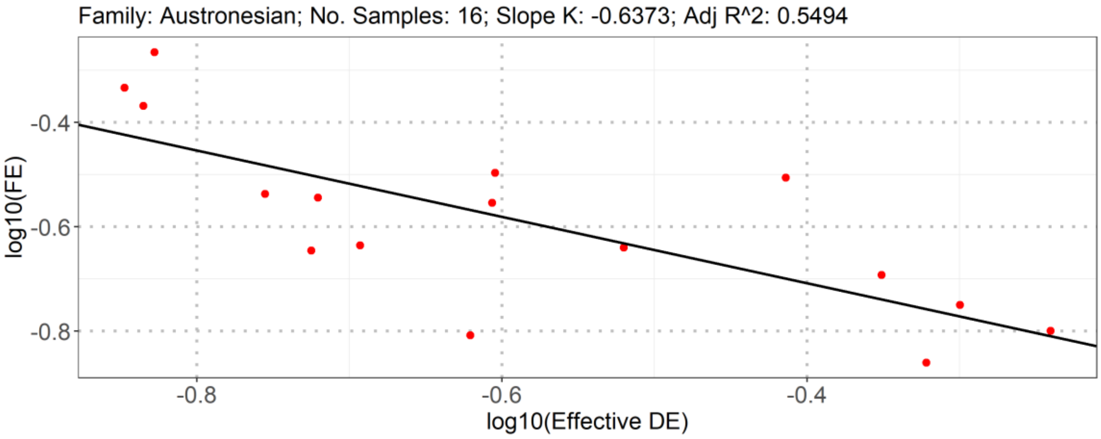

(h)

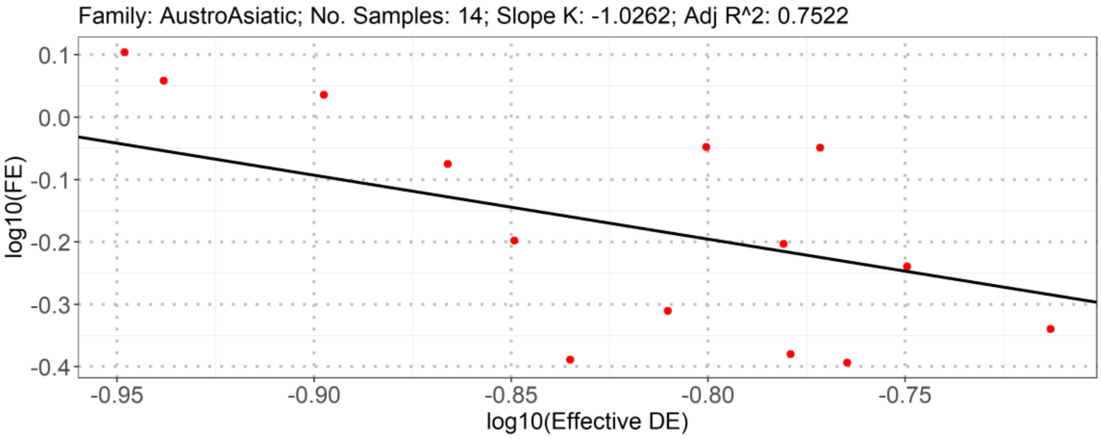

(i)

Fig S2. The linear regression plots of log-transformed (base 10) Effective DE against FE in the nine language families. The solid line in each panel denotes the best fitting linear regression curve. "Adj. R^2" is the conditional R^2 measuring the proportion of variance explained by both fixed and random factors.

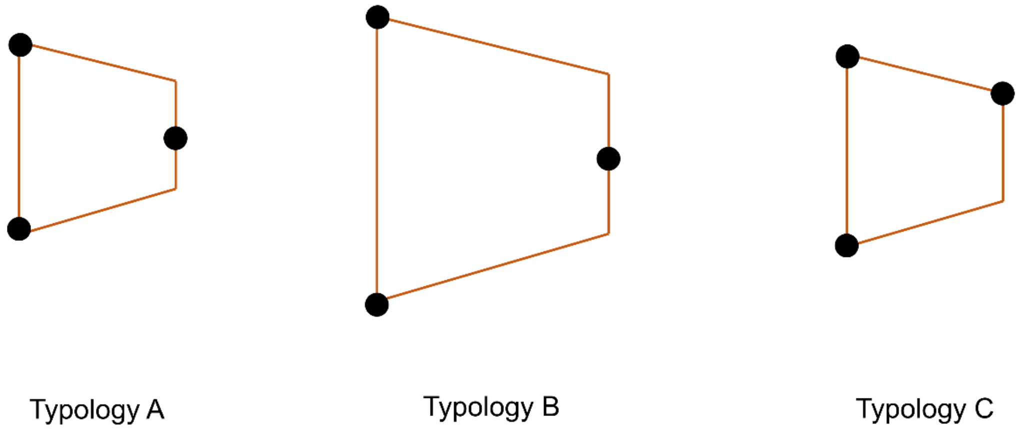

Fig S3. Three structural typologies of a vowel system having three vowels /a, i, u/. Typology A resembles Typology B in structural pattern, but both are distinct from Typology C due to a different position of a vowel.
